# A Case of Traumatic Tetanus Successfully Treated by Hot Water Baths

**Published:** 1887-09

**Authors:** T. J. Bennett

**Affiliations:** Austin, Texas


DANIEL'S
### Texas Medical Journal.

Published Monthly.—Subscription $2.00 a Year.

Vol. 3. AUSTIN, SEPTEMBER, 1887. No. 3.

### Original Articles.

CONTRIBUTED EXCLUSIVELY TO THIS JOURNAL.

*The Articles in this Department are accepted and published with the understanding that we are not responsible for, nor do we indorse the views and opinions of the writers by so doing.*

### A CASE OF TRAUMATIC TETANUS SUCCESSFULLY TREATED BY HOT WATER BATHS.

*By T. J. Bennett, M. D., Austin, Texas.*

Read before Travis County Medical Society, and, by vote of Society, ordered published in Daniel's Texas Medical Journal.

HAYWOOD FAIRCHILD, a stout, healthy, colored man, a carpenter, about 40 years old, stuck a nail into the plantar surface of his foot during the month of May of last year. The nail was immediately withdrawn. Some inflammation followed—enough to cause the patient to discontinue his avocation. During the second week after the accident, at which time the inflammation had considerably subsided, trismus set in, and rapidly the case became one of acute traumatic tetanus. General rigidity of all the voluntary muscles of the body, clonic spasms, opisthotonos, and in fact all the symptoms of a genuine case were present. Having never seen a case recover, although a few such are on record, I naturally looked forward to death as a result in this case.

I suggested the trial of hot water baths, believing and explaining that the experiment was justifiable on the grounds that whatever the result might be it could not be worse than that usually occurring, notwithstanding the most approved treatment known to the profession.

I was informed that the patient's bowels were constipated, and that he had not been able to swallow anything in the way of nourishment for a day or more before the baths were commenced. I withdrew all medicines at this time, except a laxative now and then. He had been taking a prescription of chloral and morphine before I saw him.

The patient was stripped and placed in a large bath tub filled with water as hot as could be endured. As he became accustomed to the heat, the temperature of the water was gradually elevated, until the highest possible degree of endurance compatible with safety was reached. The object was to produce relaxation. After the patient had been submerged thirty minutes, he was able to articulate distinctly, and remarked that he felt better. He was kept in the tub some twenty minutes longer, when he was thoroughly relaxed. Before removing him from the water, he swallowed a laxative and more than a pint of milk. He was then placed back in bed and wrapped up well with blankets. He fell into a quiet sleep, which lasted nearly an hour. This was the first real rest the patient had had since the inception of the attack. I felt encouraged at this, and directed that the baths be continued as often as necessary to keep the patient comfortable. For four or five days the tetanic symptoms would manifest themselves again between three and four hours after the patient had been removed from the water. The baths never failed to relieve him in from twenty to sixty minutes. Refreshing sleep usually followed.

The symptoms of the disease gradually grew milder, though it was necessary to continue the baths at irregular intervals for more than three weeks.

It is hardly necessary to say that good nourishment was kept up throughout the treatment. The patient always took his meals while in the tub. His recovery was slow, and for five or six months after he was able to be out he suffered from innumerable boils over the body.

I believe that the success in this case was due to the rest spells which the baths afforded the patient, and until we know more about the pathology of this disease, we may have to be content with merely keeping the spark of life aglow. The intervals of relaxation and rest not only allowed tired nature to recuperate and throw off the poisonous matters in the system, but they permitted nourishment to be taken and appropriated.

I would emphasize the fact that extreme and prolonged heat are necessary to success in the above plan of treatment. Symptoms of great depression mark the time of thorough relaxation, when the patient should be removed from the water. Observing this point, there can be no danger, since the stage of depression succeeds that of relaxation.

I shall not attempt to explain the exact action of hot water thus applied. I merely place the above case and mode of treatment on record because of its success.

